# High Uptake of HIV Testing in Pregnant Women in Ontario, Canada

**DOI:** 10.1371/journal.pone.0048077

**Published:** 2012-11-09

**Authors:** Robert S. Remis, Maraki Fikre Merid, Robert W. H. Palmer, Elaine Whittingham, Susan M. King, Natasha S. Danson, Lee Vernich, Carol Swantee, Carol Major

**Affiliations:** 1 Dalla Lana School of Public Health, University of Toronto, Toronto, Ontario, Canada; 2 Division of Infectious Diseases, Department of Pediatrics, The Hospital for Sick Children, Toronto, Ontario, Canada; 3 HIV Laboratory, Public Health Ontario, Etobicoke, Ontario, Canada; University of Washington, United States of America

## Abstract

In 1999, Ontario implemented a policy to offer HIV counseling and testing to all pregnant women and undertook measures to increase HIV testing. We evaluated the effectiveness of the new policy by examining HIV test uptake, the number of HIV-infected women identified and, in 2002, the HIV rate in women not tested during prenatal care. We analyzed test uptake among women receiving prenatal care from 1999 to 2010. We examined HIV test uptake and HIV rate by year, age and health region. In an anonymous, unlinked study, we determined the HIV rate in pregnant women not tested. Prenatal HIV test uptake in Ontario increased dramatically, from 33% in the first quarter of 1999 to 96% in 2010. Test uptake was highest in younger women but increased in all age groups. All health regions improved and experienced similar test uptake in recent years. The HIV rate among pregnant women tested in 2010 was 0.13/1,000; in Toronto, the rate was 0.28 per 1,000. In the 2002 unlinked study, the HIV rate was 0.62/1,000 among women not tested in pregnancy compared to 0.31/1,000 among tested women. HIV incidence among women who tested more than once was 0.05/1,000 person-years. In response to the new policy in Ontario, prenatal HIV testing uptake improved dramatically among women in all age groups and health regions. A reminder to physicians who had not ordered a prenatal HIV test appeared to be very effective. In 2002, the HIV rate in women who were not tested was twice that of tested women: though 77% of pregnant women had been tested, only 63% of HIV-infected women were tested. HIV testing uptake was estimated at 98% in 2010.

## Introduction

In 1989–91, Coates observed an HIV prevalence of 0.28 per 1,000 in pregnant women in Ontario [1]. In 1994, O'Connor reported that zidovudine prophylaxis reduced mother-infant HIV transmission by 67% [2]. In response, the Ontario Ministry of Health advised physicians to offer HIV testing to pregnant women at increased risk but test uptake remained low. In 1997, we estimated that HIV prevalence among pregnant women was substantially higher than that reported by Coates. Consequently, the Ontario Ministry of Health recommended that, beginning in January 1999, HIV counseling and testing should be offered to all pregnant women. The policy recommended an “opt-in” approach i.e. HIV testing carried out with pre-test counseling and informed consent.

To promote Ontario's new policy of prenatal HIV testing, physicians were sent pamphlets, posters and new laboratory requisitions. In September 2001, physicians who prescribed a prenatal test for hepatitis B, syphilis or rubella but not for HIV test were sent a reminder memo with the results. Letters were regularly sent to public health units including unit-specific prenatal HIV test uptake. Articles were published in the Ontario Medical Association bulletin encouraging physicians to offer prenatal HIV testing. The Toronto Public Health Department provided training to health care professionals on prenatal HIV testing. In 2003–04, posters and pamphlets aimed at women of childbearing age were sent to physicians' offices. Finally, a media campaign was aimed at Ontario's multicultural population to increase awareness of HIV in pregnancy.

We evaluated the Ontario policy in two ways. Study 1 examined HIV test uptake and HIV positivity rate among pregnant women. Study 2 was an unlinked, anonymous seroprevalence study of women who had not been tested to compare HIV rates with those tested and to determine the proportion of HIV-infected women identified by the program.

## Methods

### Ethics statement

The anonymous, unlinked seroprevalence study was approved by the University of Toronto Research Ethics Board (REB). Written consent was not obtained from women included in this analysis. The requirement for written consent was waived by the REB based on the Tri-Council Policy Statement (TCPS) guidelines [3] and the federal Canadian guidelines [4]. The TCPS guidelines provide the ethical basis for research in Canada on behalf of the three federal agencies that fund health research. These guidelines waive the requirement for written consent for studies such as ours. The conditions under which anonymous, unlinked studies can be carried out are specifically stipulated in the Canadian guidelines [4] and were adhered to in the present study.

### Data management

In Ontario, the HIV Laboratory of the Public Health Laboratory – Toronto (PHLT) and its regional laboratories perform prenatal testing for HIV, hepatitis B, rubella and syphilis. These laboratories conduct all HIV diagnostic testing in Ontario except for blood donors and life insurance applicants. Data were extracted from the PHLT information system. Most prenatal serologic testing uses a specific requisition and is managed in a dedicated database. However, some testing of pregnant women is carried out through the regular HIV diagnostic program and maintained in a separate database.

We analyzed prenatal records from January 1999 to December 2010. Women not tested according to this database were matched using name and birth date to the HIV diagnostic database to identify tests ordered through the diagnostic service. In addition to exact matches, Soundex codes allowed for spelling errors, multiple first names and last names were each treated as two separate variables (allowing linking when only one name was present) and linking with birthdates allowed for reversal when the day was 12 or less. Thus, for each woman tested in pregnancy, we were able to determine whether she was tested for HIV in either the prenatal or diagnostic program.

We used pregnancy as the unit of analysis. If a woman was tested two or more times within 258 days, we assumed the tests were performed during one pregnancy. All tests within a pregnancy were taken into account to determine HIV test status; the date assigned was that of the last test performed.

If a woman was not tested for HIV during a pregnancy (*current* test), we also examined whether she had been tested before the pregnancy (*prior* test).

### Data analysis, Study 1

Study 1 examined HIV test uptake during pregnancy. We quantified the number and proportion of pregnant women tested for HIV in either database by year, age and health region. If a woman had tested both during the current pregnancy and previously, the woman was considered to have been tested in the current pregnancy.

We also examined the number and rate of HIV infections by year, age and health region. We evaluated HIV test uptake and positivity rates by five-year age group; age was calculated as of the last test. We also calculated HIV positivity rates according public health units (PHUs) classified according their urban/rural status (five categories).

The 36 public health units (PHUs) in Ontario are in one of five health regions. However, we analyzed data using seven health regions, dividing the Central East region into Toronto and Central East, Other and the Eastern region into Ottawa and Eastern, Other. The location was based on the physician's office since data on patient residence were unavailable.

According to the Ontario HIV screening policy, women should be offered HIV testing in each pregnancy whether or not she tested previously. Therefore, we focused on testing during the current pregnancy as the most appropriate indicator of program effectiveness.

### Data analysis, Study 2

The main purpose of Study 2 was to determine the rate of infection among women not tested for HIV during prenatal care. In particular, we sought to estimate the proportion of HIV-infected women, as opposed to the total number of women, who had had a prenatal HIV test. For 2002, specimens from women who were not tested during the current pregnancy were tested anonymously on leftover sera, i.e. personal identifiers were removed. The primary analysis focused on women who had never tested. We also examined women who had tested HIV-negative previously to determine HIV incidence.

We created an unlinked data set of women tested anonymously. Data on age and health region were extracted to create an analytic file in which the serologic result was entered for each woman. We analyzed the number and rate of HIV infections by year, age and health region. We compared these results to those from women who had tested for HIV in pregnancy. Significance testing was carried out using the chi-square test. As in Study 1, the denominator was the number of pregnancies with at least one test for an infectious marker and rates were calculated per 1,000 pregnancies.

Statistical analyses were performed using SAS, Version 8.4 (Statistical Analysis Institute, Cary, NC, USA, 2004).

### Laboratory methods

Sera were tested initially by EIA and, if reactive, tested again in duplicate by EIA. If either of the repeat tests is positive, the specimen was tested by Western blot. In the unlinked study, sera were pooled in lots of 10, tested by EIA and, if a lot was reactive, each specimen was tested individually by EIA and, if repeatedly reactive, by Western blot.

### Incidence component

We measured HIV incidence among women with a previous negative HIV test. HIV incidence in 2002 was calculated both for these women who had a test in 2002 and for women who did not but were tested in the anonymous, unlinked study.

## Results

### Study 1: Prenatal HIV test uptake

**Figure 1:**
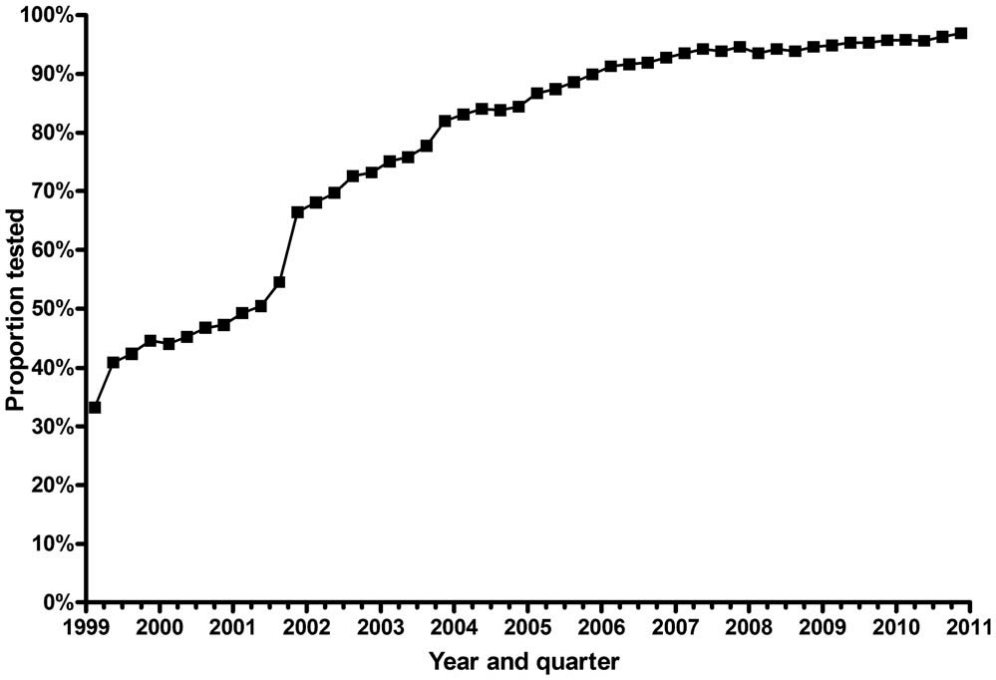
Proportion of pregnancies tested for HIV by quarter, Ontario, 1999 to 2010.

HIV test uptake increased from 40% in 1999 to 96% in 2010 (see Figure 1). The increase in test uptake was similar across age groups and health regions, though regions with lower uptake initially tended to increase more so that inter-regional variations were less in later years. Following the reminder memo implemented in September 2001, test uptake increased dramatically, from 52% in the preceding month to 62% and 66% in the two months following. Test uptake appeared to plateau somewhat in early 2004 but continued to increase gradually thereafter.

The proportion of pregnancies tested for HIV in 2010 by age and health region is presented in Table 1. HIV test uptake was lower at older ages (Χ^2^ for linear trend, p<10^−5^). HIV test uptake varied considerably across PHUs (data not shown); in 2010, test uptake by PHU varied from 92% to 99%; uptake was 95% or more in 27 of the 36 PHUs.

**Table 1:** Proportion of pregnancies tested for HIV by age group and health region, Ontario, 2010.

	Pregnancies	Tested	Proportion tested
**Overall**	**139,963**	**134,600**	**96.2%**
**Age group (years)**			
<20	6,128	5,953	97.1%
20–24	19,225	18,642	97.0%
25–29	40,393	38,940	96.4%
30–34	45,163	43,391	96.1%
35–39	23,090	22,084	95.6%
40+	5,639	5,343	94.8%
**Health region**			
Toronto	33,070	31,696	95.8%
Central East, other	38,146	36,779	96.4%
Ottawa	9,297	9,016	97.0%
Eastern, other	8,354	8,051	96.4%
Central West	26,236	25,089	95.6%
Southwest	16,300	15,718	96.4%
Northern	8,289	7,990	96.4%

Note: Information on age missing for 325 cases (0.23%) and on health region for 271 cases (0.19%).

### Study 1: HIV positivity rate

From January 1999 to December 2010, we identified 455 HIV-positive pregnant women; 268 were diagnosed for the first time during the pregnancy. Overall, 41% of HIV-positive women were previously diagnosed but this changed over time; from 1999–2004, 21% of HIV-positive women were previously diagnosed compared to 62% in 2005–10.

The new HIV positivity rate was 0.15 per 1,000 in 1999, increased subsequently to 0.34 in 2003. However, the HIV rate decreased after that and has been 0.06 to 0.13/1,000 in 2005 through 2010.

HIV rates by age and health region are presented in Table 2. The HIV positivity rate was highest among women 30–34 years of age.

**Table 2:** HIV positivity rate* by age group and health region Ontario, 2010.

	Pregnancies tested	HIV-positive	Rate per 1,000
**Overall**	**134,600**	**18**	**0.13**
**Age group (years)**			
<20	5,953	0	0.00
20–24	18,642	1	0.05
25–29	38,940	6	0.15
30–34	43,391	7	0.16
35–39	22,084	4	0.18
40+	5,343	0	0.00
**Health region**			
Toronto	31,696	9	0.28
Central East, other	36,779	7	0.19
Ottawa	9,016	0	0.00
Eastern, other	8,051	1	0.12
Central West	25,089	0	0.00
Southwest	15,718	1	0.06
Northern	7,990	0	0.00

* Rate of newly diagnosed HIV infection among women tested in 2010.

Note: Information on age was missing in 247 cases (0.18%) and on health region was missing in 261 cases (0.19%).

HIV positivity rate (per 1,000) varied by PHUs classified into five categories of urban/rural status as follows: rural 0.12, small urban 0.12, medium-sized urban 0.22, large urban/suburban 0.36, and major urban (Toronto) 0.58. The difference in positivity rates was statistically significant (p<10^−6^).

### Study 2: Unlinked anonymous study

Of the 147,411 pregnancies in 2002, 33,624 (23%) had never been tested for HIV; 21 were HIV-positive, for a crude rate of 0.62/1,000 (Table 3). The HIV positivity rate was two-fold greater than the rate of 0.31/1,000 among women in 2002 who tested for HIV, though the difference was not statistically significant. Though 77% of pregnant women were tested for HIV, only 63% of HIV-infected women were tested. In addition to the women who had never tested, we examined separately the 9,062 women who did not test in the current pregnancy but had tested HIV-negative previously. One woman was HIV-positive for a cumulative incidence rate of 0.11 per 1,000.

**Table 3:** HIV positivity by age group and health region among women ever tested and never tested, Ontario, 2002.

	Ever tested			Never tested			
	Number of pregnancies	HIV-positive	Rate per 1,000	Number of pregnancies	HIV-positive	Rate per 1,000	Relative rate
**Overall**	**113,737**	**35**	**0.31**	**33,624**	**21**	**0.62**	**2.03**
**Age group (years)**							
<20	6,269	1	0.16	1,178	0	0.00	0.00
20–24	17,740	3	0.17	4,404	4	0.91	5.37*
25–29	33,431	10	0.30	9,695	6	0.62	2.07
30–34	35,563	15	0.42	11,031	5	0.45	1.07
35–39	16,651	4	0.24	5,546	5	0.90	3.75*
40+	3,419	2	0.58	1,390	1	0.72	1.23
**Health region**							
Toronto	29,701	22	0.74	9,932	13	1.31	1.77
Central East, other	24,980	3	0.12	7,816	1	0.13	1.07
Ottawa	9,636	4	0.42	2,178	4	1.84	4.42*
Eastern, other	9,372	1	0.11	1,822	0	0.00	0.00
Central West	18,082	4	0.22	6,192	0	0.00	0.00
Southwest	14,167	1	0.07	3,813	2	0.52	7.43
Northern	7,365	0	0.00	1,777	0	0.00	-
Unknown region	434	0	0.00	94	1	10.64	-

* Statistically significant at p<0.05.

A comparison of the HIV rate among non-testers to testers is presented in Table 3. Positivity rates among the untested were higher in women 20–24 and 35–39 years of age, with relative rates of 5.37 and 3.75, respectively; both differences were statistically significant (p = 0.03 and p = 0.04, respectively). With respect to health region, non-testers in Ottawa and the Southwest region were 4.42 and 7.42 times more likely to be HIV-infected; in Ottawa, the difference was statistically significant (p = 0.04) but it was not in the Southwest region.

### Studies 1 and 2: HIV incidence

Table 4 summarizes the incidence results. Among women testing negative before 2002 and again in 2002, we observed four seroconversions, for an incidence of 0.056/1,000 person-years (py). Among women testing negative before 2002 but not tested in the context of prenatal care in 2002, we observed one seroconversion for an incidence of 0.034/1,000 py. Overall, HIV incidence was 0.050/1,000 py. Most (3/5) seroconversions were in Ottawa, where the rate was 14.7 fold greater than elsewhere (0.32 compared to 0.022/1,000 py). All five seroconversions occurred in women 30 years of age or older in whom the rate was 0.087 per 1,000 py.

**Table 4:** HIV incidence per 1,000 person-years by age group and health region among anonymous and repeat tested, Ontario, 2002.

	Anonymous			Repeat tested			All pregnancies		
	Person-years	SC*	Rate	Person-years	SC*	Rate	Person-years	SC*	Rate
**Overall**	**29,411**	**1**	**0.034**	**70,984**	**4**	**0.056**	**100,395**	**5**	**0.050**
**Age group (years)**									
<20	371	0	0.000	2,053	0	0.000	2,424	0	0.000
20–24	3,006	0	0.000	10,497	0	0.000	13,503	0	0.000
25–29	7,395	0	0.000	19,788	0	0.000	27,183	0	0.000
30–34	10,720	0	0.000	24,208	2	0.083	34,927	2	0.057
35–39	6,294	0	0.000	11,899	1	0.084	18,193	1	0.055
40+	1,620	1	0.617	2,527	1	0.396	4,147	2	0.482
Unknown age	5	0	0.000	12	0	0.000	17	0	0.000
**Health region**									
Toronto	8,286	0	0.000	16,103	1	0.062	24,389	1	0.041
Central East, other	7,321	0	0.000	16,004	0	0.000	23,325	0	0.000
Ottawa	2,720	1	0.368	6,556	2	0.305	9,276	3	0.323
Eastern, other	1,945	0	0.000	7,089	0	0.000	9,033	0	0.000
Central West	4,341	0	0.000	10,399	1	0.096	14,740	1	0.068
Southwest	2,947	0	0.000	9,797	0	0.000	12,743	0	0.000
Northern	1,743	0	0.000	4,793	0	0.000	6,536	0	0.000
Unknown region	108	0	0.000	243	0	0.000	351	0	0.000

* SC = seroconversion.

## Discussion

Our evaluation of Ontario's new HIV screening policy revealed that test uptake increased markedly, attaining 96% in 2010. Taking into account tests that did not link due to incomplete identifiers, test uptake likely attained 98% in 2010. A memo sent to physicians who did not order an HIV test appeared to be effective in helping to improve HIV test uptake, which increased 27% following its implementation. Test uptake was greatest in younger women. By 2010, the uptake rates were similar in all health regions and no public health unit was below 90%. In the 11 years from 1999 to 2010, 268 HIV-infected women were newly diagnosed, preventing at least 70 mother-infant HIV transmissions.

The anonymous, unlinked study carried out in 2002 provided valuable insights into the patterns of testing and HIV infection among non-testing women. The HIV rate among non-testers was twice that among testers; rates among 20–24 year-old and 35–39 year-old non-testers and in Ottawa were particularly high. It is possible that some HIV-positive women included in Study 2 did not test in 2002 because they knew themselves to be HIV-positive. However, women known to be HIV-positive were excluded from this analysis through record linkage. It is possible that women tested HIV-positive outside Ontario or anonymously in Ontario and therefore could not be linked. However, usually an HIV-positive woman would be retested nominally to be considered for anti-retroviral prophylaxis or treatment.

There are several other limitations to our study. We had no data on the place of residence of the women but only on the location of the prescribing physician. Some from outside the province may have traveled to Ontario for prenatal care, for example from Quebec to Ottawa; however, the number would probably be relatively small. Thus, the geographic patterns we observed may have distorted the pattern of test uptake and HIV infection by region.

The findings of our study may not necessarily be generalizable to other jurisdictions. In particular, Ontario has a universal medical care insurance program which may facilitate access to prenatal care and HIV testing. In terms of barriers to testing, however, many HIV-infected women in Ontario are from sub-Saharan Africa and the Caribbean and some members of this community may distrust the medical care system. Despite these considerations, we believe that the measures we used to achieve a high level of HIV testing, including the memo to physicians who did not order an HIV test, could be effective elsewhere.

HIV testing uptake was likely slightly higher than we observed due to our inability to match perfectly cases in the HIV diagnostic database, mainly due to HIV testing carried out without patient identifiers. Based on an analysis of the quality of identifiers available, 2–3% of HIV tests may have been missed. Thus, the actual HIV test uptake Ontario in 2010 was likely closer to 98%.

It is not possible to prove a causal relationship between the various interventions to promote HIV testing and the increase in uptake observed. However, the dramatic increase following the implementation of the memo to physicians providing prenatal care who did not prescribe an HIV test suggests that this intervention was effective.

Our study does not explain why the HIV test was not always carried out. Women may not have been tested because the test was not offered, it was offered but refused, and or because lapses could have occurred.

Although more difficult to measure, a more valid indicator of HIV screening program effectiveness would be the proportion of infected women identified, not the proportion of women screened. One UK study found that the hepatitis B infection rate among women not tested for HIV was double that of women accepting HIV testing [5], [6]. The report of Plitt et al [7] from Alberta is also instructive in this regard. She found that, in 2003–04, pregnant women who opted out of testing had a 3.3-fold higher HIV prevalence than those who opted in. In our study, we measured HIV directly among the untested using an unlinked methodology. Similar to the studies cited, we found that HIV rates were double among those not tested in 2002 and that 77% of all pregnant women were tested for HIV compared to only 63% of HIV-infected women.

Ontario's opt-in testing policy requires that HIV testing be carried out only if specifically prescribed, after counseling is provided and consent obtained. HIV test uptake in opt-in programs is generally, but not always, less than in opt-out programs, where testing is carried out on all specimens unless the patient specifically refuses. In some settings, physicians may provide HIV counseling only if the woman does not provide consent. In Canada, Alberta, Nova Scotia and Newfoundland have “opt-out” policies and testing rates in these provinces are over 95%. In Alberta, Jayarman and colleagues found that only 1.7% of women refused testing in 2000, the last year cited in their report [8].

Several studies have examined physicians practice with respect to HIV screening in Canada [9]–[18]. A study in Quebec in 1997 found that 56% of physicians reported offering HIV testing to all pregnant women [11], higher than in Ontario but still low. Gruslin and colleagues examined practice in tertiary care centre in Ottawa before and after the institution adopted a policy to offer HIV testing to all pregnant women using an opt-in approach [9]. In 1998, uptake of testing was 72% compared to 13% in 1996. In 1998, three HIV-positive women were newly identified and only 2.4% of women refused testing. Similarly, Yudin et al [19] found that an opt-out policy locally instituted in a large obstetrical practice in Toronto in 2004–05 achieved higher levels of HIV testing than were reported elsewhere in Ontario during that period.

The experience in Ontario appears to support the view that an opt-in policy can achieve high rates of prenatal screening. Nevertheless, in practice, Ontario may in fact have a mixed policy with physicians adopting an approach based on their own views and practice patterns. In fact, the distinction between the opt-in and opt-out policies may be more subtle than is often realized. Even in an opt-out policy, the pregnant woman must be informed that HIV testing is recommended and given the opportunity to refuse. In practice, this may be little different from obtaining informed consent, as required by the opt-in approach. Furthermore, the manner in which an HIV test is proposed (e.g. the duration of the discussion and the strength of the physician's recommendation to accept testing) and the woman's level of trust in her provider may be more important for test acceptance than the official policy in the province. This may explain why uptake rates are so high in some regions [9].

Mother-infant HIV transmission may occur despite an HIV-negative test early in pregnancy if the woman is infected during the pregnancy. Some have suggested that a second HIV test be considered in the third trimester [20], [21]. However, this would markedly increase the program cost and reduce its cost-effectiveness. Our analysis of HIV incidence helps shed light on this question. Assuming a second test five months after the first prenatal visit would yield about three additional HIV-positive mothers. The cost-effectiveness of both routine and selective re-testing needs to be examined. However, a study of this issue in the United States revealed that repeat testing in pregnancy would only be cost-effective if HIV incidence exceeded 1.2 per 1,000 person-years and would cost $US 819,231 per infection prevented at an incidence of 0.17 per 1,000 person-years [22]. Though the parameters related to cost in this analysis may be somewhat different in Ontario, at the HIV incidence of 0.05 per 1,000 person-years estimated in our study, it is unlikely that routine repeat testing later in pregancy would be cost-effective on Ontario.

We found a dramatic increase in prenatal HIV testing in Ontario in the 11 years following the implementation of the new screening policy, attesting to its effectiveness. Nevertheless, the results from the anonymous, unlinked component suggest that the program may not be reaching all HIV-infected women. However, this was almost nine years ago and the situation may be different now; thus, the unlinked study should be repeated. Further studies are also necessary to better understand why some women at high risk are not tested. Women who do not receive prenatal care, either because they arrive in Canada shortly before delivery or for other reasons, warrant particular attention in this regard.
